# Substantia nigra and blood gene signatures and biomarkers for Parkinson’s disease from integrated multicenter microarray-based transcriptomic analyses

**DOI:** 10.3389/fnagi.2025.1540830

**Published:** 2025-04-07

**Authors:** Hui-Hui Fan, Na-Na Hou, Dao-Lu Zhang, Dan-Ni Liu, Rong-Ting Tang, Hai-Tao Luo, Ya-Dan Song, Lei Cui, Xiong Zhang, Jian-Hong Zhu

**Affiliations:** ^1^Institute of Nutrition and Diseases and Center for Research, School of Public Health, Wenzhou Medical University, Wenzhou, Zhejiang, China; ^2^Eye and ENT Hospital of Fudan University, Shanghai, China; ^3^Institute of Geriatric Neurology and Department of Neurology, The Second Affiliated Hospital and Yuying Children’s Hospital, Wenzhou Medical University, Wenzhou, Zhejiang, China

**Keywords:** Parkinson’s disease, substantia nigra, blood, gene signature, biomarker

## Abstract

**Background:**

Parkinson’s disease (PD) is a complex, common neurodegenerative disorder with unclear etiology. The pathogenic hallmark is the death of dopaminergic neurons in the substantia nigra. PD diagnosis depends on clinical manifestation of symptoms but is lack of effective biomarker.

**Methods:**

Available human microarray-based transcriptomic datasets of the substantia nigra and blood were acquired for PD cases and controls. Robust rank aggregation and Weighted Gene Co-expression Network analysis were performed to identify gene signatures in substantia nigra and blood of PD. An overlapping analysis and validation in an independent cohort were followed to identify PD blood biomarkers.

**Results:**

Eight datasets of substantia nigra and 3 datasets of blood were retrieved, which comprised 150 substantia nigra and 571 blood samples. Integrated differentially expressed genes (DEG) and module analyses showed that the substantia nigra gene signature in PD comprised 170 key genes, mainly involved in dopaminergic synapse, neuroactive ligand-receptor interaction, calcium signaling pathway, and Parkinson disease. The blood gene signature had only 65 DEGs, but with no robust co-expression module identified. Two genes, *LRRN3* and *TUBB2A*, were both downregulated in the substantia nigra and blood of PD. But only *TUBB2A* was validated in the blood of independent cohort and showed a capacity of PD prediction.

**Conclusion:**

The present study identified PD-associated gene signatures of the substantia nigra and blood, and demonstrated that the reduced expression of *TUBB2A* in the blood is promising to predict PD. Our findings provide novel insight into the mechanisms underlying PD pathophysiology and the development of PD biomarkers.

## Introduction

Parkinson’s disease (PD) is a common and complex neurodegenerative disease pathologically characterized by the progressive death of dopaminergic neurons in the substantia nigra and the formation of α-synuclein-enriched Lewy bodies. The majority of PD is sporadic with elusive etiology, but highly likely attributes to both genetic susceptibilities and environmental exposures (Zhang et al., 2025). There are at least 90 genetic risk loci for PD as disclosed in a meta-analysis of genome-wide association studies in European ancestry (Nalls et al., 2019), with similarities and differences across different ethnicities (Zou et al., 2018; Foo et al., 2020; Pan et al., 2023). Pronounced environmental contributors include pesticides and traumatic brain injury that aggravate PD risk, and tobacco and coffee that are protective against PD (Ross et al., 2000; Ascherio et al., 2006; Thacker et al., 2007; Tanner et al., 2011; Gardner et al., 2015). Several mechanisms have been considered to be key driving PD pathogenesis and progression, including α-synuclein-centered abnormal protein misfolding and aggregation, mitochondrial dysfunction, and altered cellular trafficking and degradation (Morris et al., 2024). However, key genes that are abnormally expressed in PD and involved in these mechanisms still await further identification.

Current diagnosis of PD particularly relies on manifestations of motor symptoms including resting tremor, rigidity and bradykinesia (Bloem et al., 2021). However, about 50% of fragile dopaminergic neurons in the substantia nigra pars compacta have died by the time the symptoms can be clinically evaluated (Fearnley and Lees, 1991; Perju-Dumbrava et al., 2012), suggesting that early biomarkers are highly important for PD. Some prodromal signs such as sleep and smell dysfunctions have indeed been confirmed preceding the motor symptoms. However, regardless a variety of progresses that have been made, no effective biomarkers have been clinically proven, either by imaging, by biochemical testing in tissues or fluids, or using the prodromal signs (Bloem et al., 2021). Therefore, new efforts are still much needed in search of potential PD biomarkers.

It is always critical to understand how gene expression changes in the pathologic locus of the patients. Increased and repeated attention should be paid to such data particularly in PD because obtaining substantia nigra samples is much difficult. There have been a number of microarray-based transcriptomic datasets of the substantia nigra. We herein aimed to integrate these datasets to unveil the substantia nigra gene signature in PD for understanding new mechanisms. Meanwhile, blood may reflect certain pathological changes in the brain. We additionally acquired and analyzed microarray-based blood datasets to find blood biomarkers that reflect changes in the substantia nigra and validated them in an independent cohort.

## Materials and methods

### Microarray-based transcriptomic data extraction

Microarray-based transcriptomic datasets were searched and downloaded from Gene Expression Omnibus database^1^ by using keywords “Parkinson*” or “PD”. The searching deadline was December 15, 2023. Studies using human substantia nigra and whole blood were obtained with the following inclusion criteria: (1) including both PD cases and controls; (2) performed by microarray; (3) a minimum of total sample size for substantia nigra reached 10 samples, and for blood reached 30 samples.

### Data processing and differential analysis

Data were converted and standardized using the R (version 4.3.0). Identifications corresponding to the probe names were aligned to standard gene symbols. The converted data were further processed using the limma package of the Bioconductor package^2^. Fold changes (FCs) were calculated and sorted with log_2_FC. Robust rank aggregation^3^ was performed to integrate two or more datasets for identification of differentially expressed genes (DEGs) (Kolde et al., 2012).

### Weighted Correlation Network analysis

Weighted Correlation Network analysis (WGCNA) was performed using the R package “WGCNA” to identify gene modules that were mostly correlated with PD (Langfelder and Horvath, 2008). In detail, the function “cutreeStatic” was used to cluster samples to test the heterogeneity of the samples in the dataset. The “PickSoftThreshold” function (Parameter Settings: minimum scale-free topology fit index r^2^, 0.8) was employed to determine the best soft thresholds to identify the co-expression module. The module and sample feature correlation heatmap, and module and sample feature hierarchical clustering map were constructed to determine the key module that had the highest correlation coefficient with disease.

### Functional enrichment and protein-protein interaction analyses

Gene Ontology (GO), Kyoto Encyclopedia of Genes and Genomes (KEGG) functional enrichment, and protein-protein interaction (PPI) network analysis were performed using Metascape^4^. When the PPI network contained 3–500 proteins, the Molecular Complex Detection (MCODE) algorithm was applied to identify densely connected network components. Each MCODE component was applied with pathway and process enrichment analysis, where the best-scoring term ranked by *P*-value was retained as the functional description of the corresponding component.

### Subjects

Whole blood samples were collected from a total of 99 Han Chinese individuals, comprising 49 sporadic PD patients (28 men and 21 women, 67.1 ± 8.7 years old) and 50 controls (23 men and 27 women, 66.5 ± 8.2 years old). All subjects were from the Second Affiliated Hospital of Wenzhou Medical University. The patients were diagnosed by two movement disorder neurologists in accordance with the UK Parkinson’s Disease Society Brain Bank Criteria. Patients were assessed with the scale and recorded for disease duration. Patients with a family history of PD were excluded. Patients with Hoehn and Yahr stage ≤ 2 were considered as early PD. The control subjects were free of neurological disorders by medical history, physical and laboratory examinations. All participants provided written informed consent. The study was approved by the ethics committee of the Second Affiliated Hospital and Yuying Children’s Hospital of Wenzhou Medical University (No. LCKY201712).

### Real-time PCR

Total RNA was prepared by two times of extraction using TriPure reagent (1667165001, Roche, Indianapolis, IN, United States), and reverse-transcribed to cDNA using the PrimeScript™ RT reagent Kit (RR047A, Takara, Dalian, China). Real-time PCR was performed using a 20 μL reaction system (FastStart Essential DNA Green Master; 06924204001; Roche) in CFX Connect™ (Bio-Rad Laboratories, Hercules, CA, United States). The operating procedure was 95°C for 10 min, 40 cycles of 95°C for 10 s, 60°C for 15 s, and 72°C for 20 s, and a final extension at 72°C for 10 min. Primer pairs were 5′-CCC TTC GGC CAG ATC TTC AG-3′ and 5′-TGC CAG AGG GAA AGT GAA CA-3′ for *TUBB2A*, 5′-GCC TGC CGA CTG AGA AAA AG-3′ and 5′-GCA TGA TGC TTG AAA AGC AGT T-3′ for *LRRN3*, and 5′-TGG CAC CCA GCA CAA TGA A-3′ and 5′-CTA AGT CAT AGT CCG CCT AGA AGC A-3′ for *ACTB*. The mRNA levels were expressed as 2^–ΔCT^ following normalization to their respective *ACT*B. Levels under detection limit were given a value of zero.

### Statistical analysis

Statistical analysis was performed using the R (version 4.3.0). Visualizations of results were performed using the online platforms at^5^ ^6^. Differences in gender and age between patients and controls were assessed using χ^2^-test and *t*-test, respectively. After normality analysis using Shapiro-Wilk test, differences in mRNA expression were evaluated using Mann-Whitney *U*-test for comparisons between two groups or Kruskal-Wallis test for comparisons among three groups. Receiver-operating characteristic (ROC) curve analysis was performed to determine the specificity and sensitivity, and area under the ROC curve (AUC) was calculated as an accuracy index for evaluating the diagnostic performance. Spearman’s rank correlation coefficient was calculated to estimate the association between mRNA levels and disease duration. Values were expressed as medians with standard deviation or interquartile range. Difference was considered statistically significant when the *P*-value was < 0.05.

## Results

### Acquisition of microarray-based transcriptomic datasets

Following the inclusion criteria, a total of 11 qualified transcriptomic datasets of PD cases and controls were obtained, including 8 sets of substantia nigra and 3 sets of blood. The 8 datasets of the substantia nigra were GSE7621, GSE26927, GSE43490, GSE20163, GSE20292, GSE20333, GSE20164, and GSE49036, which contained a total of 82 PD patients and 68 controls. The datasets of the blood were GSE99039, GSE6613 and GSE72267, containing a total of 297 patients and 274 controls (Table 1).

**TABLE 1:** Summary of the GEO datasets used in the bioinformatics analyses.

Tissue type	GEO accession	Platform^1^	Year^2^	Cases	Controls	Total	Citation
Substantia nigra	GSE7621	GPL570	2007	16	9	25	Lesnick et al., 2007
	GSE26927	GPL6255	2011	12	8	20	Durrenberger et al., 2015
	GSE43490	GPL6480	2015	8	5	13	Corradini et al., 2014
	GSE20163	GPL96	2011	8	9	17	Zheng et al., 2010
	GSE20292	GPL96	2010	11	18	29	Zhang et al., 2005
	GSE20333	GPL201	2010	6	6	12	Zheng et al., 2010
	GSE20164	GPL96	2011	6	5	11	Zheng et al., 2010
	GSE49036	GPL570	2015	15	8	23	Dijkstra et al., 2015
Total				82	68	150	
Blood	GSE99039	GPL570	2017	207	233	440	Shamir et al., 2017
	GSE6613	GPL96	2006	50	22	72	Scherzer et al., 2007
	GSE72267	GPL571	2015	40	19	59	Calligaris et al., 2015
Total				297	274	571	

^1^GPL570, Affymetrix Human Genome U133 Plus 2.0 Array; GPL6255, Illumina humanRef-8 v2.0 expression beadchip; GPL6480, Agilent-014850 Whole Human Genome Microarray 4 × 44K G4112F (Probe Name version); GPL96, Affymetrix Human Genome U133A Array; GPL201, Affymetrix Human HG-Focus Target Array; GPL571, Affymetrix Human Genome U133A 2.0 Array.

^2^The year represents the time when the dataset was made public in the database.

### Transcriptomic analyses of the substantia nigra

**FIGURE 1:**
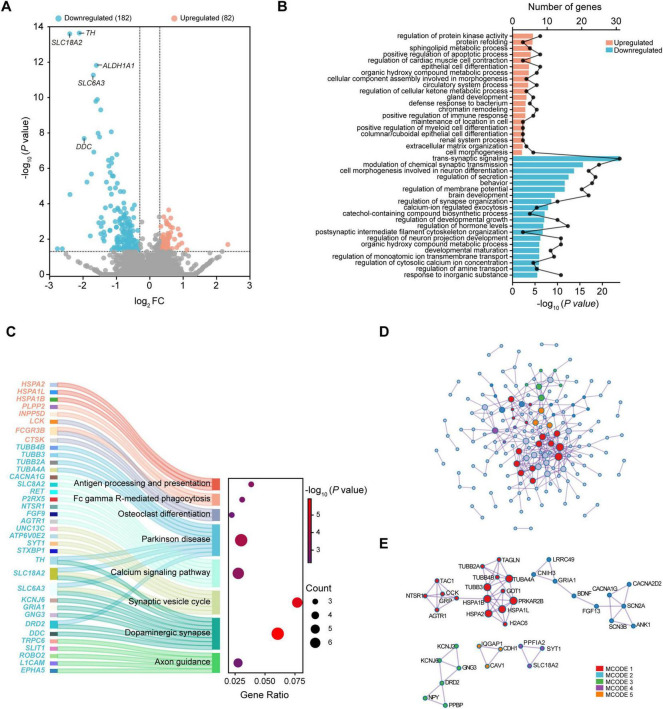
Transcriptional analysis of the substantia nigra in PD. **(A)** Volcano plot of DEGs. **(B)** Top 20 enriched biological processes of up-regulated and down-regulated DEGs. **(C)** Sankey dot plots for KEGG pathway enrichment of up-regulated and down-regulated DEGs. **(D)** Protein-protein interaction network. **(E)** Identification of MCODE components. DEGs were selected based on | log_2_FC| > 0.3 and *P*-value < 0.05. DEG, differentially expressed gene; FC, fold change; MCODE, Molecular Complex Detection; PD, Parkinson’s disease.

The threshold for robust rank aggregation was arbitrarily set at | log_2_FC| > 0.3 and *P* < 0.05. A total of 264 DEGs, including 82 up-regulated genes and 182 down-regulated genes, were acquired from the 8 datasets of substantia nigra (Figure 1A and Supplementary Table 1). Notably, almost 70% of DEGs were downregulated, among which tyrosine hydroxylase (*TH*), solute carrier family 18 member A2 (vesicular monoamine transporter 2; *SLC18A2*/*VMAT2*), solute carrier family 6 member 3 (dopamine transporter; *SLC6A3*/*DAT*), aldehyde dehydrogenase 1 family member A1 (*ALDH1A1*), and dopa decarboxylase (*DDC*) are involved in dopamine metabolism and transportation.

GO analysis showed that regulation of protein kinase activity, positive regulation of apoptotic process, positive regulation of immune response, and extracellular matrix organization were among top 20 enriched biological processes of upregulated DEGs. In contrast, downregulated DEGs were mainly enriched in trans-synaptic signaling, modulation of chemical synaptic transmission, behavior, regulation of membrane potential, calcium-ion regulated exocytosis, and regulation of cytosolic calcium ion concentration (Figure 1B). KEGG pathway enrichment analysis revealed that upregulated DEGs were significantly enriched in antigen processing and presentation, Fc gamma R-mediated phagocytosis, and osteoclast differentiation, while downregulated DEGs were enriched in Parkinson disease, calcium signaling pathway, synaptic vesicle cycle, dopaminergic synapse, and axon guidance (Figure 1C).

To analyze the interactions of DEGs and obtain the hub genes related to PD, we constructed a PPI network and obtained the subnetworks. Among the 264 DEGs, a total of 38 DEGs including 11 upregulated and 27 downregulated fitted into the PPI network complex (Figure 1D). Results showed that these DEGs were mainly enriched in the processes such as presynapse, axon, trans-synaptic signaling, anterograde trans-synaptic signaling, chemical synaptic transmission, and synaptic signaling (Supplementary Table 2). Five MCODE networks were identified (Figure 1E), where component genes were involved in pathways and processes such as protein refolding, monoatomic ion channel complex, plasma membrane protein complex, synaptic vesicle, and cell cortex (Table 2).

**TABLE 2:** Top 3 GO terms enriched in the MCODE networks.^1^

Network	GO	Description	log_10_ (*P-*value)
MCODE-1	GO:0042026	Protein refolding	–6.6
MCODE-1	GO:0005200	Structural constituent of cytoskeleton	–6.5
MCODE-1	GO:0051082	Unfolded protein binding	–6.4
MCODE-2	GO:0034702	Monoatomic ion channel complex	–9.4
MCODE-2	GO:1902495	Transmembrane transporter complex	–8.7
MCODE-2	GO:1990351	Transporter complex	–8.6
MCODE-3	GO:0098797	Plasma membrane protein complex	–5.3
MCODE-3	GO:0034765	Regulation of monoatomic ion transmembrane transport	–4.5
MCODE-3	GO:0008015	Blood circulation	–4.3
MCODE-4	GO:0008021	Synaptic vesicle	–6.4
MCODE-4	GO:0070382	Exocytic vesicle	–6.3
MCODE-4	GO:0030133	Transport vesicle	–5.5
MCODE-5	GO:0005938	Cell cortex	–6.0
MCODE-5	GO:0010038	Response to metal ion	–5.8
MCODE-5	GO:0010035	Response to inorganic substance	–5.3

^1^Ranked by *P*-values in each network. MCODE, Molecular Complex Detection.

### WGCNA and gene signature of the substantia nigra in PD

**FIGURE 2:**
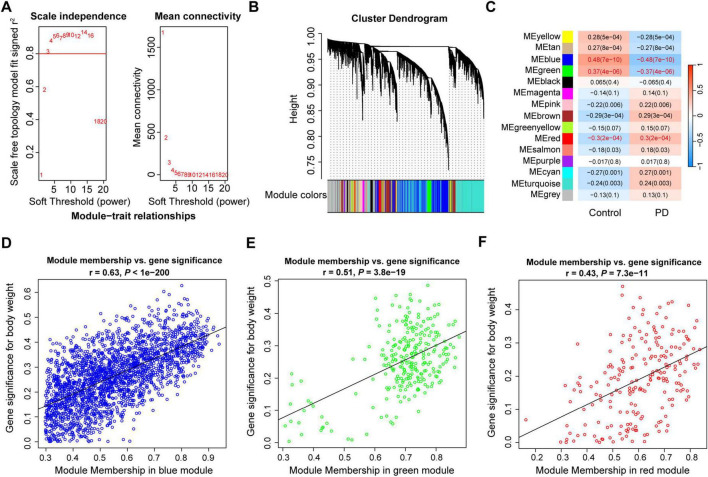
Weighted gene co-expression modules analysis of the substantia nigra in PD. **(A)** Scale free topology model fit index and mean connectivity for different soft thresholds. **(B)** Hierarchical clustering dendrogram of identified co-expressed genes in modules. **(C)** Heatmap of the correlation between module eigengenes and disease. Each cell contains a specific correlation index and *P-*value. **(D-F)** Scatter plots of module membership and gene significance in MEblue **(D)**, MEgreen **(E)** and MEred **(F)**. r, correlation coefficient; PD, Parkinson’s disease.

To explore genes closely associated with PD, WGCNA was employed to identify important modules, construct a gene co-expression network, and filter hub genes. As recommended by the WGCNA developer, using only DEGs as input before network construction can bias the results (Sanchez-Baizan et al., 2022). Each gene has a score of median absolute deviation (MAD), which is a robust statistic to quantify the variability of gene expression. Genes with higher MAD scores indicate greater variability across samples, while those with lower scores are less affected by disease or phenotype. We selected top 10,000 genes based on the MAD scores as in previous studies (Xu et al., 2022; Li et al., 2023), to ensure meaningful co-expression relationships and to reduce the noise of genes with low variability. Sample clustering analysis for quality control showed that GSM506020 was an outlier sample, and the remaining samples could be used for WGCNA workflow (Supplementary Figure 1A). The correspondence between correlation coefficients and mean connectivity under different thresholds values was measured. Results showed that the soft threshold value was 3 when the r^2^ was set at 0.8 (Figure 2A). Subsequent hierarchical clustering tree of all selected genes identified 15 distinct co-expression modules (MEs; assigned to different colors), with the largest module containing 3,280 genes (MEturquoise) and the smallest module containing 33 genes (MEcyan; Figure 2B and Supplementary Table 3). Genes that did not belong to any module were automatically assigned to the gray module. Amongst, 10 MEs were significantly correlated with PD, and MEblue, MEgreen, and MEred were top 3 correlated ones. The MEblue (*r* = –0.48, *P* = 7e-10; containing 2,221 genes) and MEgreen (*r* = –0.37, *P* = 4e-06; 268 genes) were negatively correlated with PD, while the MEred (*r* = 0.3, *P* = 2e-04; 210 genes) was positively correlated with PD (Figure 2C). Results of genetic significance for each module also supported that the MEblue, MEgreen and MEred had the highest correlation with PD (Supplementary Figure 1B).

To identified key module genes in the substantia nigra of PD patients, the correlations between modules and gene expression profiles were calculated. Results showed that the gene significance and module membership in MEblue, MEgreen, and MEred were decently correlated (Figures 2D-F). To better understand the biological pathways and processes in which the key module genes might be involved, we performed functional enrichment analysis using Metascape. Results showed that most of the biological functions were different in the genes of the three modules (Supplementary Figures 1C-E). The MEblue genes were significantly enriched in synaptic signaling, Parkinson disease, and behavior; the MEgreen genes were particularly enriched in transport, catabolism and signal transduction processes, such as autophagy, phosphatidylinositol signaling system, Golgi vesicle transport, and vesicle-mediated transport; the MEred genes were involved a variety of biological processes, such as myelination, lipid biosynthetic process, extracellular matrix organization.

**FIGURE 3:**
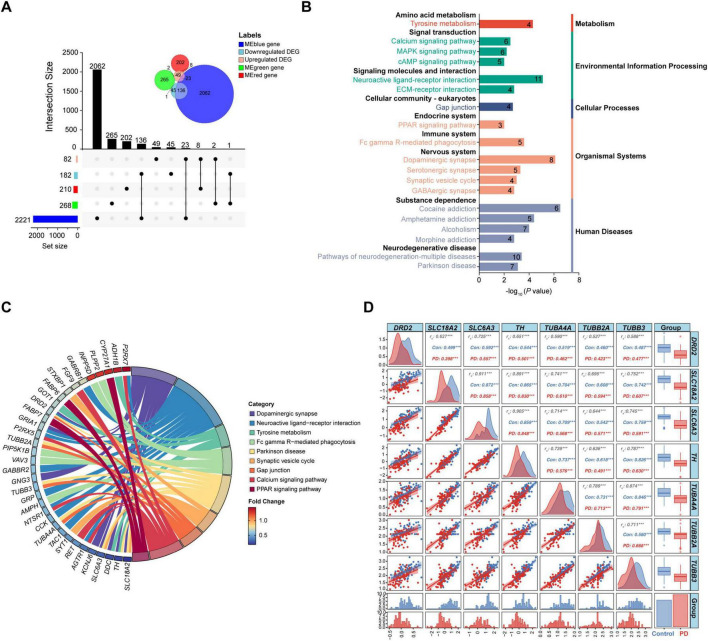
Identification of key genes in the substantia nigra of PD patients. **(A)** Upset plot and Venn diagram of numbers of common genes between DEGs and MEblue-, MEgreen-, or MEred-associated genes. **(B)** Classified KEGG pathways of the key genes. The number represents the count of enriched genes. **(C)** Chord diagram of partial enriched KEGG pathways. **(D)** Correlation analysis and distribution of the key genes in the Parkinson disease pathway. Presented are correlation matrix (upper triangle), scatter plot (lower triangle) and density plot (diagonal panel) between the key genes, histogram (bottom) and box plot (right) of data distribution of the genes, and bar chart (bottom right) of the control and PD numbers; ***P < 0.001. Con, control; DEG, differentially expressed gene; r_s_, Spearman correlation coefficient; PD, Parkinson’s disease.

Genes overlapped in the DEGs and key module genes from WGCNA were considered as the final key genes in the substantia nigra of PD. A total of 170 key genes were obtained in the substantia nigra gene signature, including 33 upregulated DEGs and 137 downregulated DEGs (Figure 3A and Supplementary Table 4). The MEblue module had most of the key genes, that is 159, while the MEred and MEgreen had only 8 and 3 key genes, respectively (Figure 3A). KEGG enrichment analysis showed that the key genes were enriched in pathways of both expected and potential novel, such as the well-known tyrosine metabolism, calcium signaling pathway, dopaminergic synapse, synaptic vesicle cycle, and Parkinson disease, and the less expected neuroactive ligand-receptor interaction, gap junction, PPAR signaling pathway, and Fc gamma R-mediated phagocytosis (Figures 3B,C). These pathways were further classified into nine subclasses under five major groups. Amongst, signaling molecules and interaction, signal transduction, and neurodegenerative disease were top 3 enriched subclasses with the largest number of enriched genes (Supplementary Table 5), suggesting an extensive dysregulation of signaling pathways. Noteworthy, *TH*, dopamine receptor 2 (*DRD2*), *SLC6A3*/*DAT*, *SLC18A2*, tubulin beta 2A (*TUBB2A*), tubulin alpha 4A (*TUBA4A*), and tubulin beta 3 (*TUBB3*) were the 7 key genes enriched in the Parkinson disease pathway (Figure 3C). The alterations in the expression profiles of these 7 genes were plotted in PD patients and controls, and Spearman correlation coefficients between expression of each gene were calculated. Results showed that the expression correlations were all reduced in PD patients compared to controls except between *TUBB2A* and *TUBB3* or *SLC6A3*, suggesting a significant dysregulation of these genes in PD (Figure 3D).

### Transcriptomic analyses and WGCNA of the blood

**FIGURE 4:**
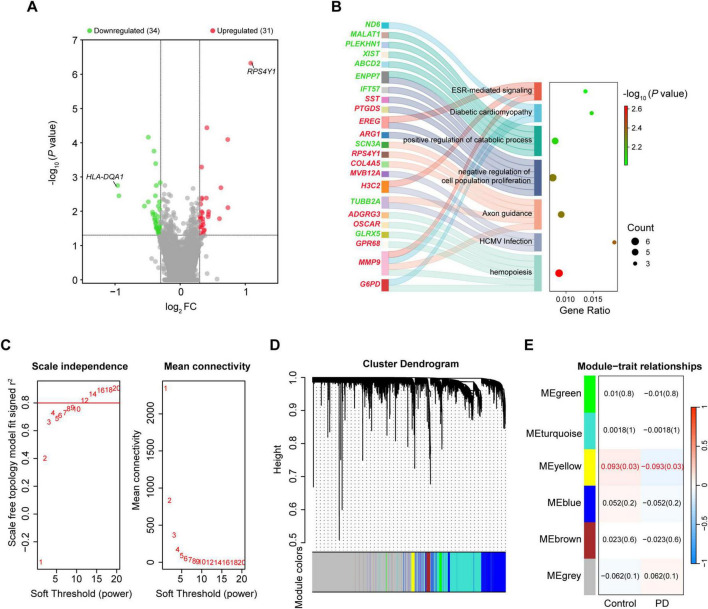
Integrated analysis of gene expression in the whole blood of PD and controls. **(A)** Volcano plot of DEGs in the blood. **(B)** Sankey dot plot of enriched KEGG pathways of DEGs. **(C)** Scale free topology model fit index and mean connectivity for different soft thresholds. **(D)** Hierarchical clustering dendrogram of identified co-expressed genes in modules. **(E)** Heatmap of the correlation between module eigengenes and disease. Each cell contains a specific correlation index and *P*-value. DEGs were selected based on | log_2_FC| > 0.3 and *P*-value < 0.05. DEG, differentially expressed gene; FC, fold change.

A total of 65 DEGs were obtained, including 31 upregulated and 34 downregulated, after integration of the 3 datasets of blood (| log_2_FC| > 0.3 and *P* < 0.05; Figure 4A and Supplementary Table 6). Ribosomal protein S4 Y-linked 1 (*RPS4Y1*) and human leukocyte antigen DQ alpha 1 (*HLA-DQA1*) were the most upregulated and downregulated genes, respectively. Annotation analysis showed that the DEGs in the blood were enriched in processes such as positive regulation of cellular catabolic process, hemopoiesis, and axon guidance (Figure 4B).

**FIGURE 5:**
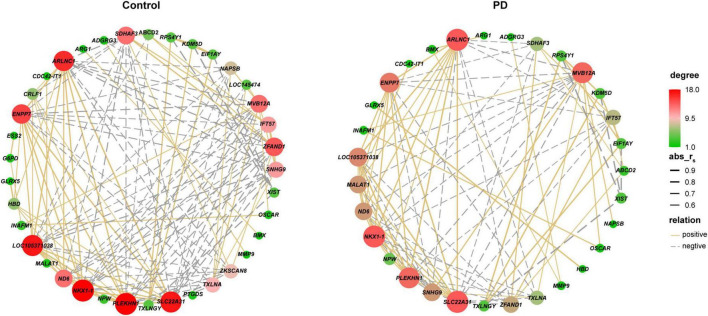
Correlation networks of DEGs in the blood of controls and PD patients. Each line has an absolute Spearman rank correlation. Degree represents the number of other nodes connected to each node. Golden lines represent positive correlation (r_s_ ≥ 0.5, *P* < 0.05); gray dotted lines represent negative correlation (r_s_ ≤ –0.5, *P* < 0.05). DEGs were selected based on | log_2_FC| > 0.3 and *P*-value < 0.05. DEG, differentially expressed gene; r_s_, Spearman correlation coefficient; PD, Parkinson’s disease.

The blood datasets were also subjected to WGCNA. Sample clustering analysis for quality control showed that GSM153508, GSM2630862 and GSM2631049 were outlier samples, and were excluded from WGCNA (Supplementary Figure 2A). The soft threshold value was 12 when the r^2^ was set at 0.8 (Figure 4C). A total of six MEs were obtained, of which only MEyellow was significantly associated with PD (Supplementary Figures 2B,C), but the correlation was weak (*r* = –0.093, *P* = 0.03; containing 173 genes; Figures 4D,E and Supplementary Table 7). Therefore, we considered all the 65 DEGs as the key genes in the blood gene signature in PD for subsequent analysis. Spearman correlation analysis revealed significant expression correlations between some of the DEGs in controls. However, these correlations were partially remodeled in PD patients, such as lost correlations between *HBD* and *PLEKHN1*, as well as new correlations between *BMX* and *MMP9* (Figure 5). This remodeling indicates that the coordinated expressions between genes are significantly disturbed in PD.

### Overlap analysis of the substantia nigra and blood key genes and validation for PD blood biomarker

**FIGURE 6:**
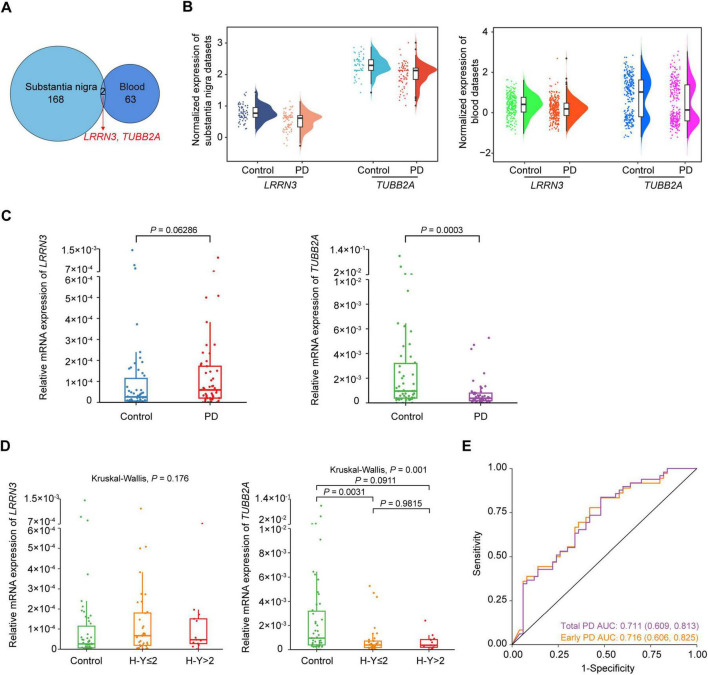
Identification of *TUBB2A* as a blood biomarker for PD. **(A)** Venn diagram of the overlapped genes between the substantia nigra and whole blood datasets. **(B)** Raincloud plot of *LRRN3* and *TUBB2A* expression in the substantia nigra and blood between PD and controls. Boxplots represent medians with interquartile range; violin diagrams represent the distributions of the data. **(C)** Expression levels of *LRRN3* and *TUBB2A* between controls and total PD patients. **(D)** Expression levels of *LRRN3* and *TUBB2A* in controls, early and late PD patients. Data are medians with interquartile range. **(E)** Prediction performance of *TUBB2A* for total and early PD. *n* = 49 total, 36 early and 13 late PD patients, and 50 controls. AUC, the area under the receiver operating characteristic curve; H-Y, Hoehn-Yahr scale; PD, Parkinson’s disease.

To analyze whether the pathological changes of gene expression in the substantia nigra were reflected in the blood, which would be good candidates for PD prediction, we performed an overlap analysis between the key genes of the substantia nigra and blood. Results revealed 2 genes, leucine rich repeat neuronal 3 (*LRRN3*) and *TUBB2A*, which were both down-regulated in PD patients (Figure 6A). The log_2_FC of *LRRN3* were –0.606 (*P* = 0.0277) and –0.401 (*P* = 0.0002), and of *TUBB2A* were –0.639 (*P* = 0.0159) and –0.489 (*P* = 0.0053), respectively in the substantia nigra and blood of PD (Figure 6B).

To validate *LRRN3* and *TUBB2A* as potential blood biomarkers for PD, we measured mRNA expression levels of these two genes in the blood of an independent cohort comprising 49 sporadic PD patients and 50 control subjects (Table 3). Age and sex of the two groups were matched (*P* > 0.05). Consistent with the above results, mRNA levels of *TUBB2A* were significantly lower (*P* = 0.0003) in the blood of PD patients than in the controls (Figure 6C). The *TUBB2A* expression was also different among early and late cases and controls (*P* = 0.001), but the difference mainly resided between cases and controls, but not between cases of different stage (Figure 6D). In contrast, *LRRN3* expression did not differ between cases and controls, or among early and late cases and controls (Figures 6C,D). As a note, the average expression of *TUBB2A* was around 48 times higher than that of *LRRN3* as shown in controls. ROC curve analysis demonstrated a decent PD prediction of *TUBB2A*, with AUC of 0.716 (95% CI, 0.606-0.825) and 0.711 (95% CI, 0.609-0.813), respectively in early and total PD (Figure 6E and Supplementary Table 8). However, no correlation was found between *TUBB2A* expression and disease duration in both early and total cases (Supplementary Figure 3).

**TABLE 3:** Demographic and clinical characteristics of PD patients and controls.

Variables	PD (*n* = 49)	Control (*n* = 50)	*P*-value
Age (years)	67.12 ± 8.70	66.5 ± 8.24	0.7157
Male, *n* (%)	28 (57.14)	23 (46)	0.3639
Hoehn and Yahr Scale	1.83 ± 0.81	NA	
Disease duration (years)	5.16 ± 3.29	NA	

Data are presented as mean ± standard deviation. PD, Parkinson’s disease.

## Discussion

**FIGURE 7:**
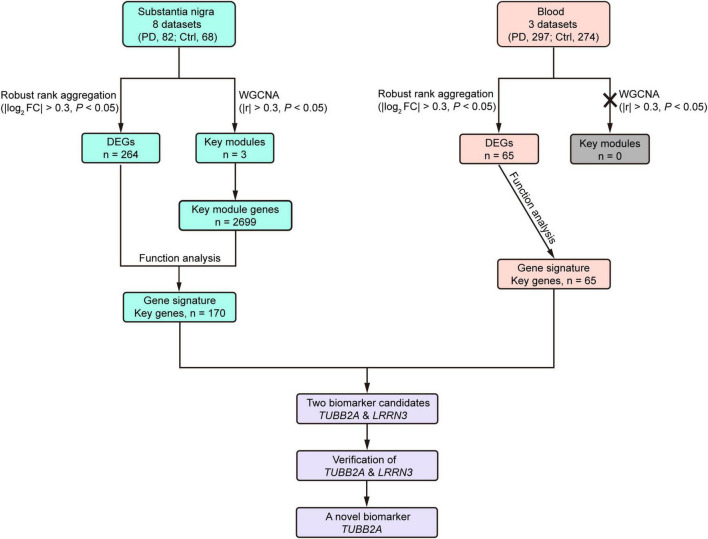
Flow chart summary of the study. Ctrl, control; DEG, differentially expressed gene; FC, fold change; PD, Parkinson’s disease; WGCNA, Weighted Correlation Network analysis.

Both molecular mechanisms and biomarkers are much needed in PD to further understand its pathophysiological processes and improve clinical diagnosis. In this study, we acquired 11 qualified microarray datasets of the substantia nigra and whole blood of PD patients and controls, which comprised 150 substantia nigra and 571 blood samples. Following data integration, DEG analysis, WGCNA and functional analyses, we identified gene signatures of the substantia nigra and blood in PD and the disturbed biological pathways, particularly in the substantia nigra. Two converged genes from the substantia nigra and blood were found and validated using an independent cohort. The reduction of blood *TUBB2A* expression could potentially be a novel biomarker for PD (Figure 7).

Integrated analysis of multi-center datasets helps to identify more reliable disease-associated driver genes. The downregulated DEGs in the substantia nigra are mainly involved in biological processes such as calcium signaling pathway, dopaminergic synapse, and trans-synaptic signaling, modulation of chemical synaptic transmission, emphasizing that disturbance of calcium homeostasis and synaptic dysfunction are key mechanisms of PD pathogenesis (Stern et al., 2022; Zhang et al., 2022). Some of the genes, including *TH*, *SLC18A2*, *SLC6A3*, *ALDH1A1*, and *DDC*, are involved in dopamine metabolism and transportation. Interestingly, the upregulated DEGs were significantly enriched in immune- and cell death-associated pathways, which are in line with the increased neuroinflammation and the loss of dopaminergic neurons in PD. In this study, gene expression datasets were from multiple independent microarray platforms, which may have differences in probe design, detection sensitivity, and genome coverage. This heterogeneity likely leads to high variations in gene expression after integration with robust rank aggregation. Therefore, we herein used the raw *P*-values to maximize the capture of DEGs, although using adjusted *P*-value is theoretically more appropriate when performing large scale expression analysis.

In addition to the DEG analysis, WGCNA is a highly robust and systematic method used in transcriptomics for revealing gene networks and hub genes connected to disease-specific modules (Langfelder and Horvath, 2008). Herein, three independent modules are disclosed to be robustly correlated with PD, two negatively and one positively. The genes in these modules may play a key role in the pathogenesis and progression of PD and are the most clinically relevant for subsequent analyses. Indeed, further analyses of DEGs and genes in these modules lead to identification of key signature in the substantia nigra of PD, which comprise a total of 170 key genes. These genes are involved in expected pathways such as dopaminergic synapse, calcium signaling pathway, and Parkinson disease, and also enriched in several potentially new pathways such as neuroactive ligand-receptor interaction, gap junction, and Fc gamma R-mediated phagocytosis. These pathways highlight that a variety of signaling molecules and interactions are disrupted in the substantia nigra during PD pathogenesis. For the genes identified in the Parkinson disease pathway, *TH*, *DRD2*, *SLC18A2*/*VMAT2*, and *SLC6A3*/*DAT* are well-known in PD for their roles in dopamine homeostasis including synthesis, signal receiving, storage and release, and reuptake (Venda et al., 2010); three tubulin genes, *TUBA4A*, *TUBB2A*, and *TUBB3*, are involved in microtubule-mediated axon outgrowth and maintenance, and vesicle trafficking (Lasser et al., 2018; Sferra et al., 2020), and mutations of these genes have been identified to cause neurological disorders (Chakraborti et al., 2016). The expression correlations between these 7 genes are mostly reduced in the patients, suggesting that both dopamine homeostasis and microtubule function are severely disturbed in PD.

Compared to those in the substantia nigra, only 65 DEGs are found in the blood, with *RPS4Y1* and *HLA-DQA1* as the most up-regulated and down-regulated genes, respectively. Interestingly, *RPS4Y1* is a Y-chromosome linked gene, while polymorphisms of *HLA-DQA1* have been reported to be associated with PD risk (Pandi et al., 2021; Yu et al., 2021). Results of WGCNA show no meaningful module correlated with PD, suggesting that the changes of gene expression in the blood do not really participate in PD pathogenesis, but more likely are certain consequences. That may explain why finding effective PD biomarkers in the blood has been difficult. Nonetheless, human blood is a feasible surrogate for brain tissue and offers unique advantages such as ease of sample collection, cost, and scalability (Lawton et al., 2020; Yazar et al., 2023). We thus analyzed whether there are changes of gene expression in the substantia nigra being reflected in the blood by real-time PCR. Generally speaking, Western blot is more appropriate to evaluate protein expression, while real-time PCR measures mRNA expression. Both are mature techniques. The sensitivity of Western blot analysis often relies on the antibody and the quantity of loading. In comparison, real-time PCR has its advantages for analyzing a large number of human blood samples, for instance, using a less volume of samples, less time-consuming, cheaper, and with higher detection sensitivity in general.

The integrated overlapping analysis, which presumably would remove noise signal and increase accuracy of targeting, indeed leads to two genes, *LRRN3* and *TUBB2A*. TUBB2A accounts for approximately 30% of all β-tubulins in human brains (Leandro-Garcia et al., 2010), and participates in the axonal transport of synaptic vesicle precursors through its interaction with kinesin (Sferra et al., 2018). LRRN3 is involved in neural development and maintenance and its polymorphisms may be associated with autism spectrum disorder (Haines et al., 2005; Sousa et al., 2010). *LRRN3* has been previously noted as a potential biomarker for PD (Guo et al., 2023). However, our independent cohort only validates that expression of *TUBB2A*, but not of *LRRN3*, is remarkably reduced in the blood of PD. Noteworthy, *TUBB2A* is relative abundantly expressed in the blood, which could be an advantage as a molecular biomarker. With AUC over 0.7, *TUBB2A* is at least a promising candidate for combined biomarker development, as it has been considered that a single form of biomarker is nearly impossible to predict PD and a combination of multiple measurements may be required for a success (Kalia and Lang, 2015).

In summary, our integrated analyses of microarray-based transcriptomes identified gene signatures of the substantia nigra and blood in PD and the associated biological processes and pathways primarily in the substantia nigra. Followed overlapping analysis and independent validation demonstrated that the reduced expression of *TUBB2A* in the blood is promising to predict PD. Our findings provide novel insight into the mechanisms underlying PD pathophysiology and the development of PD biomarkers.

## Data Availability

The original contributions presented in the study are included in the article/Supplementary material, further inquiries can be directed to the corresponding authors.

## Footnotes

1
https://www.ncbi.nlm.nih.gov/geo/

2
http://www.bioconductor.org/

3
http://cran.r-project.org/

4
http://metascape.org

5
https://www.omicstudio.cn/tool

6
https://www.bioinformatics.com.cn

## References

[B1] Ascherio A. Chen H. Weisskopf M. G. O’Reilly E. McCullough M. L. Calle E. E. (2006). Pesticide exposure and risk for Parkinson’s disease. *Ann. Neurol.* 60 197–203. 10.1002/ana.20904 16802290

[B2] Bloem B. R. Okun M. S. Klein C. (2021). Parkinson’s disease. *Lancet* 397 2284–2303. 10.1016/s0140-6736(21)00218-x 33848468

[B3] Calligaris R. Banica M. Roncaglia P. Robotti E. Finaurini S. Vlachouli C. (2015). Blood transcriptomics of drug-naive sporadic Parkinson’s disease patients. *BMC Genomics* 16:876. 10.1186/s12864-015-2058-3 26510930 PMC4625854

[B4] Chakraborti S. Natarajan K. Curiel J. Janke C. Liu J. (2016). The emerging role of the tubulin code: From the tubulin molecule to neuronal function and disease. *Cytoskeleton* 73 521–550. 10.1002/cm.21290 26934450

[B5] Corradini B. R. Iamashita P. Tampellini E. Farfel J. M. Grinberg L. T. Moreira-Filho C. A. (2014). Complex network-driven view of genomic mechanisms underlying Parkinson’s disease: Analyses in dorsal motor vagal nucleus, locus coeruleus, and substantia nigra. *Biomed. Res. Int.* 2014:543673. 10.1155/2014/543673 25525598 PMC4261556

[B6] Dijkstra A. A. Ingrassia A. de Menezes R. X. van Kesteren R. E. Rozemuller A. J. Heutink P. (2015). Evidence for immune response, axonal dysfunction and reduced endocytosis in the substantia nigra in early stage Parkinson’s disease. *PLoS One* 10:e0128651. 10.1371/journal.pone.0128651 26087293 PMC4472235

[B7] Durrenberger P. F. Fernando F. S. Kashefi S. N. Bonnert T. P. Seilhean D. Nait-Oumesmar B. (2015). Common mechanisms in neurodegeneration and neuroinflammation: A BrainNet Europe gene expression microarray study. *J. Neural Transm.* 122 1055–1068. 10.1007/s00702-014-1293-0 25119539

[B8] Fearnley J. M. Lees A. J. (1991). Ageing and Parkinson’s disease: Substantia nigra regional selectivity. *Brain* 114 2283–2301. 10.1093/brain/114.5.2283 1933245

[B9] Foo J. N. Chew E. G. Y. Chung S. J. Peng R. Blauwendraat C. Nalls M. A. (2020). Identification of risk loci for Parkinson disease in Asians and comparison of risk between Asians and Europeans: A genome-wide association study. *JAMA Neurol.* 77 746–754. 10.1001/jamaneurol.2020.0428 32310270 PMC7171584

[B10] Gardner R. C. Burke J. F. Nettiksimmons J. Goldman S. Tanner C. M. Yaffe K. (2015). Traumatic brain injury in later life increases risk for Parkinson disease. *Ann. Neurol.* 77 987–995. 10.1002/ana.24396 25726936 PMC4447556

[B11] Guo X. Hu W. Gao Z. Fan Y. Wu Q. Li W. (2023). Identification of PLOD3 and LRRN3 as potential biomarkers for Parkinson’s disease based on integrative analysis. *NPJ Parkinsons Dis.* 9:82. 10.1038/s41531-023-00527-8 37258507 PMC10232497

[B12] Haines B. P. Gupta R. Jones C. M. Summerbell D. Rigby P. W. (2005). The NLRR gene family and mouse development: Modified differential display PCR identifies NLRR-1 as a gene expressed in early somitic myoblasts. *Dev. Biol.* 281 145–159. 10.1016/j.ydbio.2005.01.030 15893969

[B13] Kalia L. V. Lang A. E. (2015). Parkinson’s disease. *Lancet* 386 896–912. 10.1016/S0140-6736(14)61393-3 25904081

[B14] Kolde R. Laur S. Adler P. Vilo J. (2012). Robust rank aggregation for gene list integration and meta-analysis. *Bioinformatics* 28 573–580. 10.1093/bioinformatics/btr709 22247279 PMC3278763

[B15] Langfelder P. Horvath S. (2008). WGCNA: An R package for weighted correlation network analysis. *BMC Bioinformatics* 9:559. 10.1186/1471-2105-9-559 19114008 PMC2631488

[B16] Lasser M. Tiber J. Lowery L. A. (2018). The role of the microtubule cytoskeleton in neurodevelopmental disorders. *Front. Cell. Neurosci.* 12:165. 10.3389/fncel.2018.00165 29962938 PMC6010848

[B17] Lawton M. Baig F. Toulson G. Morovat A. Evetts S. G. Ben-Shlomo Y. (2020). Blood biomarkers with Parkinson’s disease clusters and prognosis: The oxford discovery cohort. *Mov. Disord.* 35 279–287. 10.1002/mds.27888 31693246 PMC7028059

[B18] Leandro-Garcia L. J. Leskela S. Landa I. Montero-Conde C. Lopez-Jimenez E. Leton R. (2010). Tumoral and tissue-specific expression of the major human beta-tubulin isotypes. *Cytoskeleton* 67 214–223. 10.1002/cm.20436 20191564

[B19] Lesnick T. G. Papapetropoulos S. Mash D. C. Ffrench-Mullen J. Shehadeh L. de Andrade M. (2007). A genomic pathway approach to a complex disease: Axon guidance and Parkinson disease. *PLoS Genet.* 3:e98. 10.1371/journal.pgen.0030098 17571925 PMC1904362

[B20] Li D. Yang H. Lyu M. Wang J. Xu W. Wang Y. (2023). Acupuncture therapy on dementia: Explained with an integrated analysis on therapeutic targets and associated mechanisms. *J. Alzheimers Dis.* 94 S141–S158. 10.3233/JAD-221018 36776063 PMC10473135

[B21] Morris H. R. Spillantini M. G. Sue C. M. Williams-Gray C. H. (2024). The pathogenesis of Parkinson’s disease. *Lancet* 403 293–304. 10.1016/S0140-6736(23)01478-2 38245249

[B22] Nalls M. A. Blauwendraat C. Vallerga C. L. Heilbron K. Bandres-Ciga S. Chang D. (2019). Identification of novel risk loci, causal insights, and heritable risk for Parkinson’s disease: A meta-analysis of genome-wide association studies. *Lancet Neurol.* 18 1091–1102. 10.1016/S1474-4422(19)30320-5 31701892 PMC8422160

[B23] Pan H. Liu Z. Ma J. Li Y. Zhao Y. Zhou X. (2023). Genome-wide association study using whole-genome sequencing identifies risk loci for Parkinson’s disease in Chinese population. *NPJ Parkinsons Dis.* 9:22. 10.1038/s41531-023-00456-6 36759515 PMC9911746

[B24] Pandi S. Chinniah R. Sevak V. Ravi P. M. Raju M. Vellaiappan N. A. (2021). Association of HLA-DRB1, DQA1 and DQB1 alleles and haplotype in Parkinson’s disease from South India. *Neurosci. Lett.* 765:136296. 10.1016/j.neulet.2021.136296 34655711

[B25] Perju-Dumbrava L. D. Kovacs G. G. Pirker S. Jellinger K. Hoffmann M. Asenbaum S. (2012). Dopamine transporter imaging in autopsy-confirmed Parkinson’s disease and multiple system atrophy. *Mov. Disord.* 27 65–71. 10.1002/mds.24000 22102521

[B26] Ross G. W. Abbott R. D. Petrovitch H. Morens D. M. Grandinetti A. Tung K. H. (2000). Association of coffee and caffeine intake with the risk of Parkinson disease. *JAMA* 283 2674–2679. 10.1001/jama.283.20.2674 10819950

[B27] Sanchez-Baizan N. Ribas L. Piferrer F. (2022). Improved biomarker discovery through a plot twist in transcriptomic data analysis. *BMC Biol.* 20:208. 10.1186/s12915-022-01398-w 36153614 PMC9509653

[B28] Scherzer C. R. Eklund A. C. Morse L. J. Liao Z. Locascio J. J. Fefer D. (2007). Molecular markers of early Parkinson’s disease based on gene expression in blood. *Proc. Natl. Acad. Sci. USA.* 104 955–960. 10.1073/pnas.0610204104 17215369 PMC1766335

[B29] Sferra A. Fattori F. Rizza T. Flex E. Bellacchio E. Bruselles A. (2018). Defective kinesin binding of TUBB2A causes progressive spastic ataxia syndrome resembling sacsinopathy. *Hum. Mol. Genet.* 27 1892–1904. 10.1093/hmg/ddy096 29547997

[B30] Sferra A. Petrini S. Bellacchio E. Nicita F. Scibelli F. Dentici M. L. (2020). TUBB variants underlying different phenotypes result in altered vesicle trafficking and microtubule dynamics. *Int. J. Mol. Sci.* 21:1385. 10.3390/ijms21041385 32085672 PMC7073044

[B31] Shamir R. Klein C. Amar D. Vollstedt E. J. Bonin M. Usenovic M. (2017). Analysis of blood-based gene expression in idiopathic Parkinson disease. *Neurology* 89 1676–1683. 10.1212/WNL.0000000000004516 28916538 PMC5644465

[B32] Sousa I. Clark T. G. Holt R. Pagnamenta A. T. Mulder E. J. Minderaa R. B. (2010). Polymorphisms in leucine-rich repeat genes are associated with autism spectrum disorder susceptibility in populations of European ancestry. *Mol. Autism* 1:7. 10.1186/2040-2392-1-7 20678249 PMC2913944

[B33] Stern S. Lau S. Manole A. Rosh I. Percia M. M. Ben Ezer R. (2022). Reduced synaptic activity and dysregulated extracellular matrix pathways in midbrain neurons from Parkinson’s disease patients. *NPJ Parkinsons Dis.* 8:103. 10.1038/s41531-022-00366-z 35948563 PMC9365794

[B34] Tanner C. M. Kamel F. Ross G. W. Hoppin J. A. Goldman S. M. Korell M. (2011). Rotenone, paraquat, and Parkinson’s disease. *Environ. Health Perspect.* 119 866–872. 10.1289/ehp.1002839 21269927 PMC3114824

[B35] Thacker E. L. O’Reilly E. J. Weisskopf M. G. Chen H. Schwarzschild M. A. McCullough M. L. (2007). Temporal relationship between cigarette smoking and risk of Parkinson disease. *Neurology* 68 764–768. 10.1212/01.wnl.0000256374.50227.4b 17339584 PMC2225169

[B36] Venda L. L. Cragg S. J. Buchman V. L. Wade-Martins R. (2010). alpha-Synuclein and dopamine at the crossroads of Parkinson’s disease. *Trends Neurosci.* 33 559–568. 10.1016/j.tins.2010.09.004 20961626 PMC3631137

[B37] Xu Z. Zhou T. Wang Y. Zhu L. Tu J. Xu Z. (2022). Integrated PPI- and WGCNA-retrieval of hub gene signatures for soft substrates inhibition of human fibroblasts proliferation and differentiation. *Aging (Albany NY)* 14 6957–6974. 10.18632/aging.204258 36057261 PMC9512501

[B38] Yazar V. Dawson V. L. Dawson T. M. Kang S. U. (2023). DNA methylation signature of aging: Potential impact on the pathogenesis of Parkinson’s disease. *J. Parkinsons Dis.* 13 145–164. 10.3233/JPD-223517 36710687 PMC10041453

[B39] Yu E. Ambati A. Andersen M. S. Krohn L. Estiar M. A. Saini P. (2021). Fine mapping of the HLA locus in Parkinson’s disease in Europeans. *NPJ Parkinsons Dis.* 7:84. 10.1038/s41531-021-00231-5 34548497 PMC8455634

[B40] Zhang F. Liu Y. X. Zhu Y. Y. Yu Q. Y. Msigwa S. S. Zeng Z. H. (2025). Epidemiologic risk and prevention and interventions in Parkinson disease: From a nutrition-based perspective. *J. Nutr.* S0022-3166 35–35. 10.1016/j.tjnut.2025.01.028 39900185

[B41] Zhang J. Shen Q. Ma Y. Liu L. Jia W. Chen L. (2022). Calcium homeostasis in Parkinson’s disease: From pathology to treatment. *Neurosci. Bull.* 38 1267–1270. 10.1007/s12264-022-00899-6 35727497 PMC9554109

[B42] Zhang Y. James M. Middleton F. A. Davis R. L. (2005). Transcriptional analysis of multiple brain regions in Parkinson’s disease supports the involvement of specific protein processing, energy metabolism, and signaling pathways, and suggests novel disease mechanisms. *Am. J. Med. Genet. B Neuropsychiatr. Genet.* 137B 5–16. 10.1002/ajmg.b.30195 15965975

[B43] Zheng B. Liao Z. Locascio J. J. Lesniak K. A. Roderick S. S. Watt M. L. (2010). PGC-1alpha, a potential therapeutic target for early intervention in Parkinson’s disease. *Sci. Transl. Med.* 2:52ra73. 10.1126/scitranslmed.3001059 20926834 PMC3129986

[B44] Zou M. Li R. Wang J. Y. Wang K. Wang Y. N. Li Y. (2018). Association analyses of variants of SIPA1L2, MIR4697, GCH1, VPS13C, and DDRGK1 with Parkinson’s disease in East Asians. *Neurobiol. Aging* 68 159.e7–159.e14. 10.1016/j.neurobiolaging.2018.03.005. 29622492

