# Supplementary material for: Substantia nigra and blood gene signatures and biomarkers for Parkinson’s disease from integrated multicenter microarray-based transcriptomic analyses
Source: Front Aging Neurosci. 2025 Apr 7;17:1540830. doi: 10.3389/fnagi.2025.1540830 (PMC12009882; doi:10.3389/fnagi.2025.1540830)
Supplement: Supplementary file 2 [file Image_1.pdf]

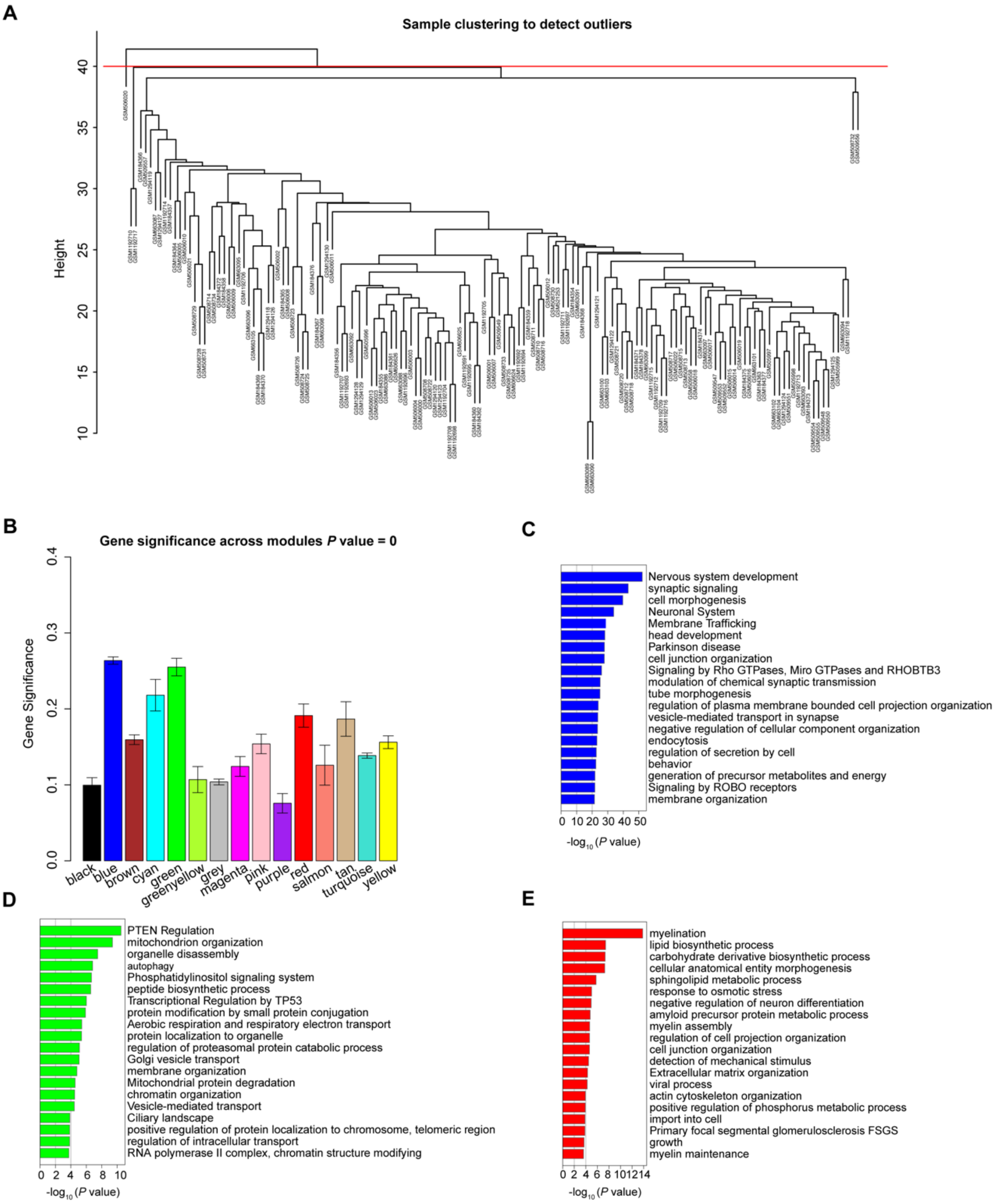

Supplementary Figure 1. Construction of Weighted Gene Co-Expression Network in the substantia nigra. A Sample clustering for outlier samples. **B** Gene significance of different modules. **C**-**E** Functional enrichment analyses of the genes in MEblue (**C**), MEgreen (**D**) and MEred (**E**), respectively. Top 20 clusters with their representative enriched terms (one per cluster) were listed. The heatmap was ranked by *P* values.

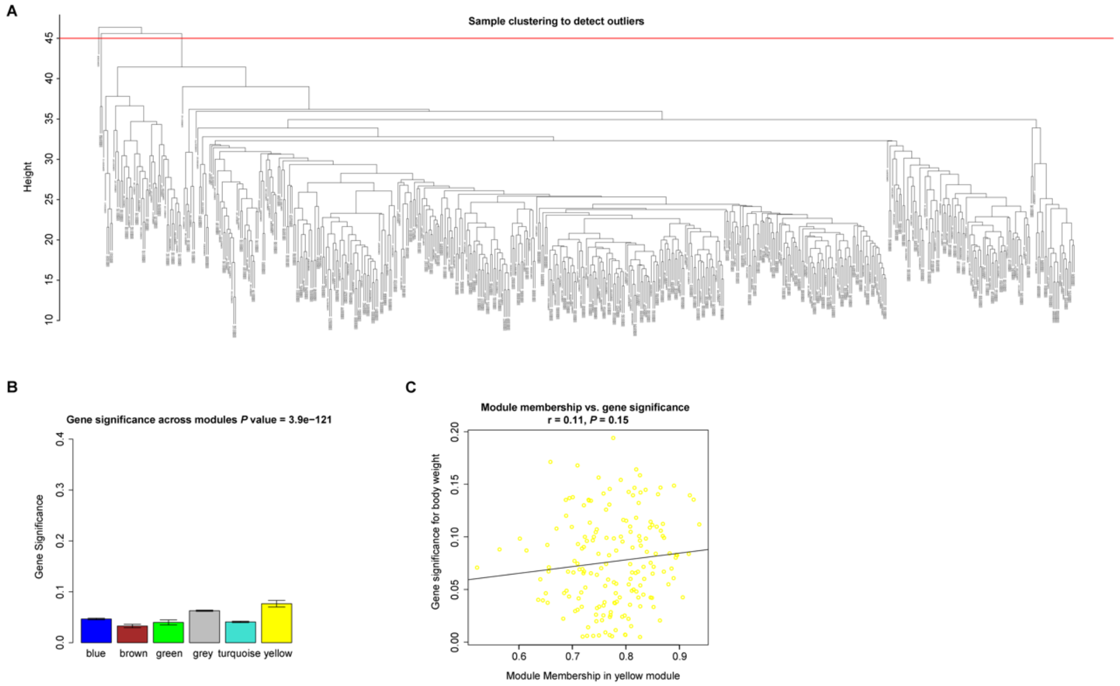

Supplementary Figure 2. Construction of Weighted Gene Co-Expression Network in the blood. A Sample clustering for outlier samples. **B** Gene significance of different modules. **C** Scatter plot of module membership and gene significance in yellow modules.

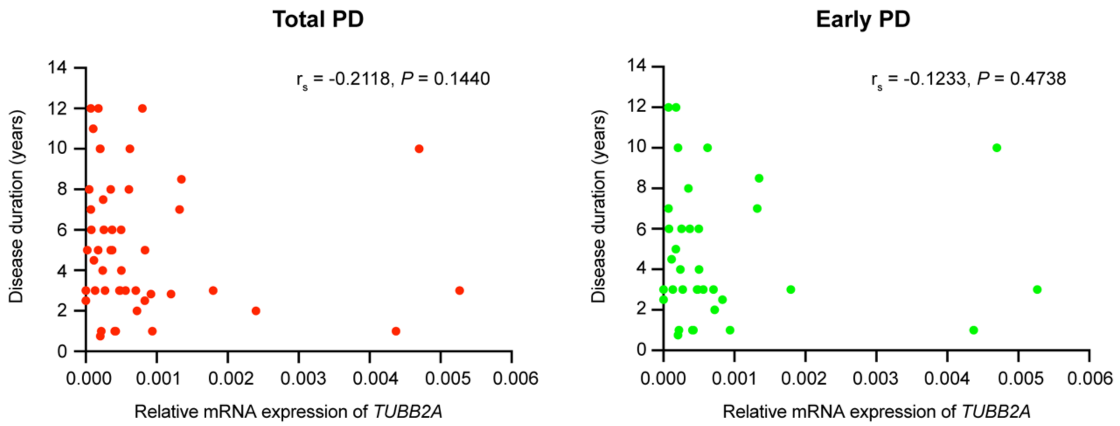

Supplementary Figure 3. Correlation analyses between the blood *TUBB2A* expression and Hoehn-Yahr scale in total and early PD patients. n = 49 total and 36 early PD patients.
